# Genetic and epigenetic landscape of early-onset oral squamous cell carcinoma: Insights of genomic underserved and underrepresented populations

**DOI:** 10.1590/1678-4685-GMB-2024-0036

**Published:** 2024-08-05

**Authors:** Daniela Adorno-Farias, Sebastián Morales-Pisón, Guilherme Gischkow-Rucatti, Sonia Margarit, Ricardo Fernández-Ramires

**Affiliations:** 1 Chilean Hereditary Cancer Group (GCCH), Santiago, Chile.; 2 University of Chile, School of Dentistry, Oral Medicine and Pathology Department, Santiago, Chile.; 3 Mayor University, School of Medicine and Health Sciences, Santiago, Chile.; 4 Desarrollo University, ICIM, School of Medicine, Santiago, Chile.

**Keywords:** Genetic, epigenetic, young patients, oral squamous cell carcinoma.

## Abstract

Oral squamous cell carcinoma (OSCC) has a poor prognosis and the treatment employed generates significant physical deformity in patients. In recent years, an increase in the incidence of cases of OSCC has been observed in adult patients up to 45 years old in several genetic underrepresented and underserved countries. The increase in OSCC cases in young people is very relevant because it shows that OSCC does not make exceptions and hereditarily must play an important role. This fact has not been associated with an evident biological basis, and a large majority of these patients do not present the classic principal risk factors association. OSCC is the result of accumulation of genetic and epigenetic alterations and this information is still fragmented in the literature, mainly in the young group. Conducting studies with a comprehensive analysis of genetic and epigenetic data is crucial, to provide greater understanding of the underlying biology of OSCC, because this information can be decisive to determine targets for therapeutic treatment. We review the main germline and somatic aspects of genetic and genomic variation in OSCC considering the absence of genomic data from developing countries such as Chile and the rest of Hispano-America.

Introduction

Oral cancer is a disease with expressive and growing international relevance, and different aspects have contributed to this condition: a) higher incidence; b) referral of patients for treatment at late stages; c) significant morbidity and d) 5-year survival rate below 50%. This cancer represents the 18th most common malignancy worldwide, with almost 380,000 (2%) new cases and 178,000 (1.8%) new deaths estimated in 2020 (Sung et al., 2021; Barsouk et al., 2023) (Figure 1). The incidence rates of oral cancer are highest in South and Southeast Asia, Central and Eastern Europe and South America (Barsouk *et al*., 2023). Brazil, being the largest country in the region, often serves as a focal point for oral squamous cell carcinoma (OSCC) research, reporting a considerable burden of cases attributed to factors such as tobacco use, alcohol consumption, and socioeconomic disparities. In Latin America, the percentages of new cases and deaths from oral cavity and lip cancer estimated for 2020 are slightly lower, at 1.3 and 1.2, respectively (Ferlay et al., 2021). However, discrepancies have been reported between estimates of new cases of lip and oral cavity cancers reported by GLOBOCAN for Latin America and the Caribbean when compared with data from the Brazilian National Cancer Institute. These discrepancies highlight a probable underreporting of oral cancer cases in Latin America and the Caribbean. (Santos-Silva et al., 2024). Brazil, Argentina, and Uruguay report the highest incidence rates for this cancer (Curado et al., 2016). Cuba and the Dominican Republic also contribute significantly to the oral cancer rates in Latin America (Freire et al., 2021).

**Figure 1-:**
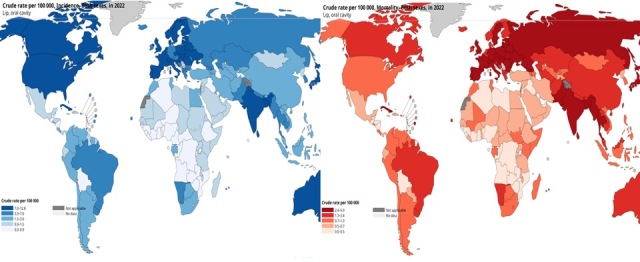
Incidence (blue) and mortality (red) rates of OSCC worldwide (GLOBOCAN Today Cancer; https://gco.iarc.who.int/today; Ferlay *et al.*, 2024).

OSCC represents over 90% of cases of malignant neoplasms of the oral cavity. This neoplasm mostly affects men over 50 years old and is mainly located on the tongue and floor of the mouth. However, in recent years, an increase in the incidence of cases has been demonstrated in adult patients up to 45 years old, in the USA, Europe, and other countries ( Ferreira e Costa et al., 2022; Jones et al., 2022; Kolegova et al., 2022), accounting for a total of 3.1% to 18.8% of cases (dos Santos Costa et al., 2018), and this fact has not been associated with an evident biological basis (Jones *et al.*, 2022). Many literary references indicating an increase in the incidence of OSCC in young patients were developed in the United States using the SEER database (Ferreira e Costa *et al.*, 2022). Individuals diagnosed with OSCC up to the age of 45 have been considered young patients according to the literature (Oliver et al., 2019; Jones *et al.*, 2022), although some studies have reported the cutoff point for this category up to 40 years (Oliver *et al.*, 2019; Ferreira e Costa *et al.*, 2022). OSCC is of paramount significance in young patients due to its unique epidemiological and clinical characteristics. While traditionally associated with older age groups, the rising incidence of OSCC among young individuals has become a compelling area of study. This shift raises concerns about distinct etiological factors and aggressive biological behavior in younger patients, prompting a need for tailored diagnostic and therapeutic strategies even with the study of oral potentially malignant disorders (Pennacchiotti et al., 2021; Adorno-Farias et al., 2023). This complex relationship has not yet been clarified at the molecular level. Last year a study described for the first time 13 genes that are found in OED in a Latin American population, of which five genes have already been observed in oral squamous cell carcinoma (Adorno-Farias *et al.*, 2023) (Figure 2).

**Figure 2 -:**
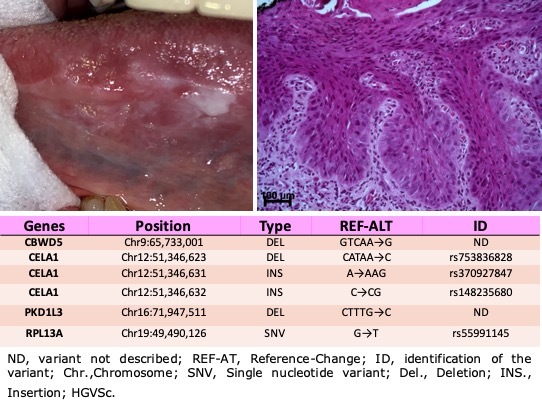
Representative figure of the clinical, histopathological characteristics, and information of six variants observed in a Brazilian male patient with leukoerythroplasia on lateral border of the tongue associated with high-grade epithelial dysplasia. (Data observed in a previous study conducted by our group. Clinical and histopathological figures previously published in ^*Adorno-Farias et al. (2023*^ ^).^

The increasing prevalence of risk factors such as human papillomavirus (HPV) infection and changing lifestyle habits underscores the urgency to understand OSCC’s dynamics in this demographic. Moreover, early-onset OSCC may exhibit different clinical manifestations and treatment responses compared to cases in older populations (Ferreira e Costa et al., 2022). Exploring the molecular and genetic underpinnings of OSCC in young patients is crucial for refining prognostic markers and developing targeted interventions. The study of OSCC in the younger cohort not only addresses a growing public health issue but also contributes valuable insights that can potentially reshape preventive measures, screening protocols, and treatment approaches to optimize outcomes for this specific age group (Satgunaseelan et al., 2021).

It has generally been established that a classic association of a history of alcohol and tobacco consumption are the main risk factors for OSCC (Kolegova et al., 2022). In fact, 70% of OSCC patients consume tobacco and 36% consume alcohol. However, the prevalence of these habits is more associated with older than young patients (Kolegova *et al.*, 2022). The role of tobacco consumption is controversial among young patients, but a total of 41-65% of non-smokers have been reported in this patient group (Batistella et al., 2022; Ferreira e Costa et al., 2022; Jones et al., 2022; Kolegova *et al.*, 2022). In fact, there has also been an increase in cases in women under 40 years of age who have not been exposed to tobacco or alcohol (Atula et al., 1996; O’Regan et al., 2006; dos Santos Costa et al., 2018; Miranda Galvis et al., 2018). In a study conducted in India, 88% of OSCC patients up to the age of 35 who do not consume tobacco have been reported (Kuriakose et al., 1992). In recent decades, human papillomavirus (HPV) has emerged as a risk factor for SCC, however, current data is not reliable to associate HPV with OSCC in patients younger than 45 years old (Kolegova *et al.*, 2022). 

OSCC is the result of accumulation of genetic and epigenetic alterations, which lead to the activation of proto-oncogenes and inactivation of multiple tumor suppressor genes (Eljabo et al., 2018). Historically, in relation to classical OSCC (related to patients older than 50 years), it is mentioned that there is a greater opportunity for the development of tumors in the oral cavity due to exposure to various environmental mutagens (carcinogens) (Eljabo *et al.*, 2018). Mutagens create fields with genetically or epigenetically altered cells that have a higher risk of undergoing malignant transformations.

Seven potential driver genes have been identified for OSCC: *TP53, CDKN2A, CASP8, NOTCH1, FAT1, ATXN1, CDC42EP1* (Campbell et al., 2021; Kolegova et al., 2022)*. TP53* is the most frequently mutated gene in OSCC and mutations in this gene have been frequently associated with tobacco and alcohol exposure (Kolegova *et al.*, 2022). *TP53* has been shown to have a central node in the interaction network between somatic and germline altered genes (Cury et al., 2021). On the other hand, it has been reported that *TP53* mutations are less common in young, non-smoking patients with tongue OSCC than in young smoking patients and other OSCC patients (Kolegova *et al.*, 2022). Regarding *FAT1*, it has been found that inactivation of this gene is not necessary for the development of OSCC in young patients (Kolegova *et al.*, 2022). A recent study was able to identify for the first time a lower tumor mutation burden and *EGFR* amplification with an associated increase in RNA abundance in patients with OSCC younger than 50 years old (Satgunaseelan et al., 2021). 

Somatic alterations

The study at hand (Campbell et al., 2021) represents a pioneering endeavor, constituting the most extensive examination to date of the somatic mutational landscape of oral tongue squamous cell carcinoma (OTSCC). In scrutinizing 227 specimens from eight diverse sources, including 107 early-onset cases, their analysis identified seven putative driver genes for OTSCC, unveiling two hitherto unreported genes-*ATXN1* and *CDC42EP1*. Impressively, 82.8% of specimens exhibited missense or truncating mutations in at least one of these seven genes, with *TP53* emerging as the most frequently mutated (63.0%). Another study from MD Anderson (Cancer Discovery Project), shows *TP53* as the most mutated gene in OSCC patients (Figure 3A), being R175H and R110L the most recurrent mutations (Figure 3B). Intriguingly, recurrent mutations, predominantly within tumor suppressor genes, displayed a mutually exclusive pattern, suggesting their pivotal roles in OTSCC carcinogenesis. Surprisingly, early-onset OTSCC manifested significantly fewer non-silent mutations compared to typical-onset counterparts, even after adjusting for overall tobacco use, although no significant associations were discerned between putative driver genes and age of OTSCC onset (Campbell *et al.*, 2021).

**Figure 3-:**
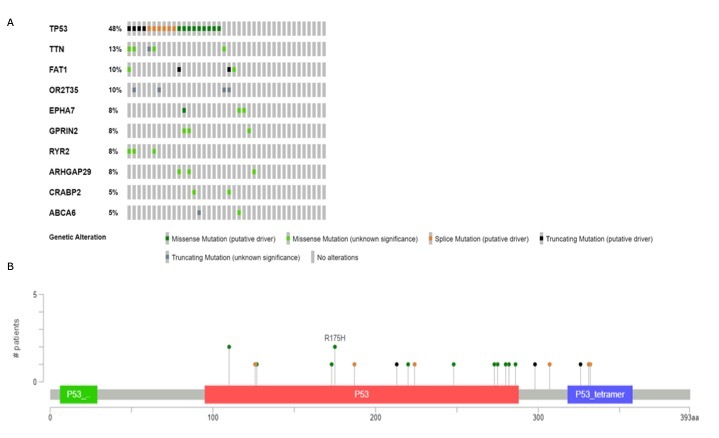
Top 10 genes most frequently mutated in the Cancer Discovery OSCC cohort from MD Anderson (A). Most recurrent mutation in *TP53* gene from the same cohort (B). cBioPortalData (https://www.cbioportal.org/; Cerami *et al.*, 2012; Gao *et al.*, 2013).

Among the seven identified putative OTSCC driver genes, *TP53, NOTCH1, CDKN2A, FAT1*, and *CASP8* have previously been implicated in head and neck squamous cell carcinoma (HNSCC). *TP53*, renowned as the most frequently mutated gene in non-human papillomavirus (HPV) or smoking-related HNSCC and OSCC, exhibited comparable non-silent mutation rates in early-onset (62.6%) and typical-onset (62.5%) OTSCC specimens. Recurrent *TP53* mutations, particularly at residues R248, R273, and R175, mirrored their prevalence in human cancer and HNSCC. Notably, while p.R175H is deemed a high-risk mutation in HNSCC, it displayed no association with age of onset in the study (Campbell et al., 2021).

Functionally, *TP53* operates as a tumor suppressor gene orchestrating diverse downstream pathways involved in metabolism, cell-cycle regulation, DNA repair, and apoptosis (Olivier et al., 2002). Paradoxically, a study proposed that recurrent mutations in *TP53* confer gain-of-function activities, transforming these genes into oncogenes rather than tumor suppressors (Campbell et al., 2021). Demonstrated in multiple studies, such mutations (R175H, R273H, and R248Q) drive invasive tumor growth and resistance to chemotherapeutic agents, challenging the traditional characterization of *TP53* in carcinogenesis (Olivier M *et al.*, 2002; Campbell *et al.*, 2021).

*CDKN2A*, universally inactivated in non-HPV-related HNSCC, displayed variable mutation rates in our study, akin to earlier reports in oral cavity cancer (Lawrence et al., 2015). *NOTCH1*, a participant in the squamous differentiation Notch pathway acting as a tumor suppressor in oral cavity carcinogenesis (Pickering et al., 2013), exhibited similar mutation rates in oral cavity SCC (Pickering *et al.*, 2013) and in the OTSCC cohort (Campbell et al., 2021), though disruptions in the Notch pathway were absent (Pickering *et al.*, 2013). Previous genomic analyses proposed four major driver pathways in OSCC-*TP53*, Notch, cell cycle (*CDKN2A, CCND1*), and mitogenic signaling (*EGFR, HRAS, PI3K*) (Pickering *et al.,* 2013). A recent study verified three of these pathways as prime drivers in OTSCC, notably excluding the mitogenic signaling pathway (Campbell *et al.*, 2021). *FAT1* and *CASP8*, prominent in OSCC, were identified as potential driver genes in OTSCC, albeit infrequently co-occurring (Campbell *et al.*, 2021).

Novel driver genes *ATXN1* and *CDC42EP1* were unraveled, their roles in OTSCC and OSCC heretofore unreported (Campbell et al., 2021). *ATXN1*, encoding a chromatin-binding protein, exhibited time-dependent functions in cancer development, promoting mitosis during early tumorigenesis and enhancing invasion during hypoxic tumor growth (Tong et al., 2011). Eight of nine specimens with *ATXN1* mutations were associated with advanced-stage disease, suggesting involvement in epithelial-mesenchymal transition and cancer metastasis (Campbell *et al.*, 2021). *CDC42EP1*, a member of the Rho GTPase family, represents a novel player in cancer development, particularly in OTSCC (Campbell *et al.*, 2021).

Despite lacking experimental validation, *ATXN1* and *CDC42EP1* are postulated as cancer driver genes in a subset of OTSCC. Their mutual exclusivity with *NOTCH1* mutations and the recurrence of specific deletions support their potential significance. Strikingly, our study suggests a genetic distinction between early-onset and typical-onset OTSCC, contradicting previous claims of genomic similarity. Despite the study’s sizable genomic cohort, limitations, including varied sequencing platforms and data processing, demand cautious interpretation. The prevalence of advanced-stage specimens raises concerns about passenger mutations, emphasizing the need for a deeper understanding of molecular progression. Limitations in tobacco use and HPV status data necessitate further investigations into their roles in OTSCC. Despite these constraints, this study significantly advances our comprehension of OTSCC’s genetic landscape, laying the groundwork for future diagnostic and therapeutic endeavors (Campbell et al., 2021).

Most of these young patients lack many of the classic risk factors and characteristics of other head and neck cancers, including tobacco and alcohol exposure, HPV-positive status, and *TP53* mutations (Maroun et al., 2021). Some studies have reported hereditary factors that are associated with the development of HNSCC in young adults. Early-onset malignancies generally have a genetic determination, especially in cases where there are no associated environmental factors (Kolegova et al., 2022). Studies have found polymorphic variants and other genetic factors that are more common in young HNSCC patients. It has been reported that the KIR2DL1+ -HLA-C2+ genotype is exclusively associated with HNSCC patients under 55 years of age (Dutta et al., 2014). In addition, a recent study has identified a germline *CDKN2A* mutation in a 39-year-old patient with tongue HNSCC, without significant associated risk factors and with a history of HNSCC in her mother. This may suggest that germline mutations in this gene increase the risk of developing squamous cell carcinomas of the head and neck (Jeong et al., 2022).

In 2018, a study published the first evidence suggesting that genetic factors play a role in the etiology of oral cavity carcinomas in 30 HPV-negative young patients (Fostira et al., 2018). Using a panel of 94 cancer predisposition genes, the authors reported that 13.3% of patients had germline variants characterized by loss of function in *CDKN2A, SDHB,* and *RECQL4* (Fostira *et al.*, 2018). Then, another study also found germline variants in *CDKN2A* and *RECQL4* in patients with HPV-negative OSCC (Cury et al., 2021). A recent study identifies germline variants in DNA repair genes and *FAT1* that potentially contribute to a higher risk of developing the disease at a young age (Cury *et al.*, 2021). Additionally, they suggest that germline variants in DNA repair genes could contribute to better survival, while germline alterations in *FAT1* are associated with worse survival (Cury *et al.*, 2021).

Germline susceptibility

Familial and hereditary cancers refer to conditions where an individual has a heightened risk of developing cancer due to genetic factors within their family. Familial cancer implies an increased occurrence of cancer cases within a family, possibly influenced by shared environmental and lifestyle factors. In contrast, hereditary cancer specifically results from inherited genetic mutations that predispose individuals to an elevated risk of developing certain types of cancer. These mutations are passed down from one generation to the next and can significantly increase the likelihood of cancer onset. Individuals with hereditary cancer syndromes often have family histories featuring multiple cases of the same or related cancers. Genetic testing and counseling are crucial tools for identifying hereditary cancer risks, helping individuals make informed decisions about preventive measures and screening. Understanding familial and hereditary aspects of cancer aids in tailoring personalized healthcare strategies and early interventions for at-risk individuals.

The majority of head and neck cancers (HNCs) cases are sporadic, with less than 3% having familial connections (Mroueh et al., 2020). Mroueh *et al.* (2020) determined the fraction of HNC cases exhibiting familial clustering and assessed the relative risk of HNC for family members and spouses of patients diagnosed with early-onset HNC. This indicates that, at most, only a small proportion of HNCs is primarily due to inherited genetic mutations. First- or second-degree relatives of patients diagnosed with early-onset HNC (≤40 years old) did not show an increased relative risk of HNC or other malignancies compared to the general population. The cumulative incidence of HNC for first-degree relatives of early-onset HNC patients is less than 0.10% by age 40 and 0.43% at any age. These findings stand in contrast with some case-control studies reporting an elevated risk of HNC in subjects with a family history of the disease. Notably, this study focused on early-onset HNC, allowing for a more precise investigation of potential inherited factors. The results aligned with certain studies that did not find a strong association between family history of HNC and cancer risk, particularly when considering lifestyle factors such as tobacco and alcohol consumption. The reduced risk of HNC observed in spouses of probands raises questions, and the study suggests potential explanations, such as low-risk lifestyle behaviors adopted by spouses after the proband’s diagnosis. However, confounding variables like smoking habits and HPV status are not adjusted for, and the definition of spouse lacks a specific time period (Mroueh *et al.*, 2020).

Cury et al. (2021) explored the mutational landscape of HNSCC in young adults was, focusing on genetic factors rather than traditional risk factors such as alcohol, tobacco, and HPV. The research, encompassing 45 cases, revealed that 90% of the patients exhibited at least one of these risk factors, but the mutational profiles did not distinctly correlate with these exposures. Instead, genetic factors, particularly germline variants, were identified as significant contributors to HNSCC development in young individuals. Notably, germline alterations in genes like *CDKN2A, RECQL4*, and DNA repair genes (e.g., *ACD, TPP1, RTEL1, TERT*) were prevalent. The study also detected somatic mutations in known cancer driver genes such as *TP53, CDKN2A, FAT1, NOTCH1, and PIK3CA* (Cury *et al.*, 2021). The findings suggested a substantial impact of germline variants in DNA repair genes on HNSCC susceptibility and better overall survival. Conversely, germline alterations in the *FAT1* gene were associated with worse survival outcomes. The integration of germline and somatic data revealed *TP53* as a central player connecting these genetic events. The study, despite limitations in sample size, provided a comprehensive genetic profile of HNSCC in young patients, emphasizing the potential significance of genetic factors in understanding and managing the disease (Cury *et al.*, 2021).

Recently, Brake et al. (2023) underscored the significance of identifying pathogenic germline variants (PGVs) in patients with head and neck cancer, revealing that 10.5% of such patients had PGVs, mostly undetectable through standard clinical genetic testing. The lack of widely-practiced standards for germline testing in head and neck cancer contributes to a high “miss” rate (95%) when testing is not performed. Current guidelines for germline testing in this context are limited to five syndromes, mostly associated with cancers beyond the head and neck. The study’s findings emphasized the importance of expanding genetic testing criteria, given that only one patient out of 21 PGV carriers met existing guidelines. The implications extend to familial cascade testing, as demonstrated in a case where clinical action was taken based on detected PGVs, highlighting the need for more comprehensive and specific guidelines in the field of hereditary head and neck cancer (Brake *et al.*, 2023).

Epigenetic modifications

Although genetic alterations play a role in OSCC development, epigenetics explains how gene expression is regulated without altering the DNA sequences (Vatsa et al., 2023). Epigenetic changes involve DNA and histone modifications that are not encoded in the DNA sequence, although these changes are not hereditary (Egger et al., 2004). Epigenetic changes include DNA methylation, histone modification, chromosomal remodeling, and microRNA dysregulation, and these play a significant role in OSCC development (Kolegova et al., 2022). DNA methylation appears to be the most important, with hypermethylation observed in OSCC (Lingen et al., 2011; Nikitakis et al., 2018 a ; Rapado-González et al., 2024). Abnormal distribution of DNA methylation is one of the most studied epigenetic changes in cancer, where global hypomethylation leading to the activation of oncogenes and transposons is often accompanied by focal hypermethylation of CpG islands in the promoter region of tumor suppressor genes, leading to transcriptional silencing (Kulis and Esteller, 2010). Recent studies have reviewed molecules involved in the DNA damage response mechanism, as dysfunction of this mechanism may be associated with malignant transformation, mainly observing an association between increased expression of γH2AX, the phosphorylated form of one of the most common histones (H2AX), and the possibility of malignant transformation (Nikitakis *et al.*, 2018b; Zhu et al., 2018). The hypermethylation of CpG islands in the promoters of *RASSF1A, RASSF2A, MGMT, DAPK,* and *FHIT* genes is an early event in OSCC and is considered a potential marker for early cancer diagnosis (D’Souza and Sarannath, 2017). In 2022, Rapado-González *et al.* (2024) published the first genome-wide study on DNA methylation in OSCC of tongue, which identified a group of new tumor-specific DNA methylation markers that have diagnostic potential in saliva: *A2BP1, ANK1, ALDH1A2, GFRA1, TTYH1, and PDE4B*. 

In 2023, Inchanalkar et al. (2023) presented a comprehensive analysis of genome-wide methylation profiles associated with oral potentially malignant disorder (leukoplakia) and gingivobuccal complex cancers (GBC-OSCC). The research identified a methylation signature specific to leukoplakia and GBC-OSCC, offering potential for identifying high-risk precancerous lesions with a tendency for malignant transformation. The integration of methylation data with genomic copy number and transcriptomic data revealed 32 genes with prognostic significance, indicating their regulation by both copy number alterations (CNA) and methylation. In leukoplakia and GBC-OSCC, genome-wide DNA methylation analysis identified differentially methylated positions (DMPs), with leukoplakia showing 846 DMPs (303 hypomethylated, 543 hypermethylated) and GBC-OSCC showing 5111 DMPs (3127 hypomethylated, 1984 hypermethylated). The methylation profiles in leukoplakia and tumors differ from normal tissues, and aberrations increase as the lesions progress. The study identifies 45 hypermethylated promoters common between leukoplakia and tumors, with known tumor suppressor genes (TSGs) *CDKN1B, ZFP82, SHISA3, GPX7*, and *IRF8* among them. Seven potential oncogenes associated with poor survival, including *FAT1* and *GHR*, exhibited hypomethylation and amplification-dependent upregulation. Notably, *FAT1*, previously associated with both tumor suppression and oncogenic roles, is revealed to be regulated by promoter hypomethylation, potentially contributing to its oncogenic role in GBC-OSCC. Prognostic biomarkers associated with survival outcomes revealed copy number loss in *CASP4* and gain in *ISG15*. Interestingly, gain in *ISG15* is associated with better relapse-free, disease-specific, and overall survival, making it a potential prognostic marker for GBC-OSCC. Loss of *CASP4* is associated with poor relapse-free survival (Inchanalkar *et al.,* 2023).

Regarding microRNAs, dysregulation of some of these has been observed in OSCC, affecting the proliferation, apoptosis, differentiation, and migration of tumor cells. Let-7c, miR-130a-3p, miR-361-5p, miR-99a-5p, miR-29c-3p, and let-7d-5p are overexpressed in aggressive tongue OSCC in patients under 30 years compared to non-aggressive tumors in older patients (Hilly et al., 2016). Regarding epigenetic alterations in young patients, current data is scarce, and additional studies using high-throughput transcriptomic and proteomic methods are required to establish a complete picture of the molecular events that trigger and govern OSCC in young adults (Kolegova et al., 2022). A deep understanding of epigenetic modifications may allow the development of new diagnostics and therapies for the effective management of OSCC in young people. Additionally, it is feasible that the methylation of tumor suppressor gene promoter regions may promote tumorigenesis through the alteration of signaling pathways in patients diagnosed with young onset OSCC.

The epigenetic and genetic alterations were initially thought to be discrete mechanisms driving the tumor, but whole exome sequencing of various cancers has revealed the interdependence of epigenetic and genetic alterations (Vatsa et al., 2023). To date, efforts to undertake whole exome analysis of OSCC in young patients remains fragmented. Next generation sequencing (NGS) accelerates the process of studying DNA and many types of RNA by generating digital and quantifiable data that can be mapped back to the genome. NGS has revolutionized our understanding of carcinogenesis and cancer care in the last decade (Satgunaseelan et al., 2021). Findings from NGS studies of oral lesions will help us better understand the genetic aspects of a tumor traditionally considered environmental. Increasingly, genetic changes are being identified that can lead to carcinogenesis at a younger age or after relatively low exposure to carcinogens, in a variety of malignancies (Satgunaseelan *et al.*, 2021). For example, melanomas in young patients with low cumulative exposure to solar ultraviolet radiation are 2.7 times more likely to show mutations in the *BRAF* gene (proto-oncogene B-Raf) (Bauer et al., 2011). These findings suggest that malignancies that occur at a younger age and in the absence of conventional cancer risk factors may harbor different genetic profiles compared to older patients (Satgunaseelan *et al.*, 2021). Several genome-wide DNA methylation studies have been conducted in head and neck squamous cell carcinomas; however, there are few global DNA methylation analyses in OSCC (Rapado-González et al., 2024), regardless of age. It is important to note that these genetic and epigenetic changes may be targets for therapeutic treatment.

The molecular basis and prognosis of early-onset OSCC in young patients is still controversial (Oliver et al., 2019; Ferreira e Costa et al., 2022). Given the high frequency of *NOTCH1* mutations in early-onset squamous cell carcinoma of the oral cavity and oropharynx, Notch signaling pathway inhibitors that have shown efficacy in other types of cancer could be considered as an additional therapeutic approach in treating young patients with this cancer (Kolegova et al., 2022). Some studies suggest that OSCC in patients under 45 years of age exhibits more aggressive behavior and worse prognosis compared to older patients (Adduri et al., 2014; Panda et al., 2022). A recent study observed, after a follow-up of 29.4 months, that young adult patients under 45 years of age with OSCC in the tongue who did not consume alcohol or tobacco demonstrated higher rates of locoregional recurrence and distant metastasis than patients over 45 years old (Jones et al., 2022). However, current studies have demonstrated survival superiority in young patients (Omura et al., 2023) while others have reported no significant difference in the clinical behavior and prognosis of OSCC in different patient groups (Miranda Galvis et al., 2018). The treatment of young patients does not differ from that used in older patients, which includes surgery followed or not by radiation or chemotherapy (Kolegova *et al.*, 2022).

Although highly prevalent like other types of malignant neoplasms, OSCC shows a negative trend attributed to several factors, such as late diagnosis, field cancerization, and inherent biological aggressiveness (tendency for invasive growth and metastatic invasion to lymph nodes) (Nikitakis et al., 2018a). On the other hand, the increase in OSCC cases in young people is very relevant because it shows that OSCC does not make exceptions. The current prevention of OSCC is summarized in early detection and prevention programs that encourage a decrease in tobacco consumption. However, classic etiological factors such as tobacco, alcohol, and HPV do not appear to be associated with young patients. Available current data indicate that OSCC in young people may be a distinct clinical entity, and there is a need to identify diagnostic and prognostic markers and therapeutic targets for effective treatment of this pathology (van der Kamp et al., 2022). Current information regarding genetic and epigenetic alterations of OSCC in young patients remains fragmented, and more data is required to better understand the causes and molecular drivers that can serve as the basis for suggesting effective treatment algorithms.

Genetic counseling and germline testing for early-onset HNC

Genetic counseling (GC) in cancer is a specialized, evidence-based practice aimed at assisting individuals and families in understanding the role of genetic factors in cancer susceptibility and risk. This process involves the integration of medical, familial, and psychosocial information to provide comprehensive risk assessment and facilitate informed decision-making regarding genetic testing and subsequent medical management. Genetic counselors, typically trained healthcare professionals such as biologists and nurses with expertise in medical genetics and counseling, play a pivotal role in interpreting complex genetic information, elucidating potential hereditary cancer risks, and addressing psychological and ethical aspects of genetic testing (Manrique et al., 2013; Schienda and Stopfer, 2020). Through a thorough evaluation of family and medical histories, genetic counselors identify individuals at increased risk for hereditary cancer syndromes, guiding them through the implications of genetic testing results. Additionally, they educate patients about available risk reduction strategies, personalized screening protocols, and, when applicable, potential interventions for at-risk family members. This collaborative and patient-centered approach empowers individuals to make informed choices, promoting proactive healthcare and enabling the implementation of tailored strategies for cancer prevention, early detection, and personalized treatment based on their unique genetic profiles (Laurino et al., 2018).

For familial breast and ovary cancer syndromes it is well established the risk assessment and management for patients and their families but it is not for HNCs (Birkeland et al., 2016). For example, testing rates varies among cancer types, with higher rates observed for primarily *BRCA1/2*-related cancers (26.0% for breast, 38.6% for ovarian) compared to Lynch syndrome-associated types (5.6% for colorectal, 6.4% for endometrial). Despite similar pathogenic frequencies in Lynch syndrome and *BRCA1/2* genes, under-testing persisted in Lynch syndrome-linked cancers. Traditionally, the criteria for genetic testing selection have relied on factors such as clinical presentation, tumor characteristics, and family history, as outlined in established practice guidelines. These guidelines presently advocate testing for individuals at high risk, considering personal or family history, age at diagnosis, and specific disease-related risk factors (van der Kamp et al., 2022). However, the existing criteria are primarily derived from studies focused on individuals of Northern European descent, and there is a limited amount of data from populations that have been historically underrepresented in clinical research. As a result, the effectiveness and sensitivity of these criteria in racially and ethnically diverse populations remain unexplored. Considering the highest prevalence of HNC in Latins and in Asians, compared to other ethnics, and the that investigations have predominantly focused on white European cohorts gathered from academic medical institutions, registry cohorts, and genetic testing enterprises, it is imperative to design a specific methodology to select high-risk patients prone to be carriers of germline pathogenic or likely-pathogenic variants. Clinical and family management must be discussed in a multidisciplinary way considering oral pathologists, geneticists, genetic counselors, and oncologists (Brake et al., 2023).

Conclusions

The increase of OSCC cases in young people in genetic underserved and underrepresented countries is a current worldwide issue and most scientific articles reporting these data are less than 5 years old. Most studies conducted on young patients with OSCC have been carried out in the USA, leaving many unanswered questions regarding other countries in America. Despite the low frequency of OSCC compared to other types of malignancies, this neoplasm has a poor prognosis and the treatment employed generates significant physical deformity in patients, significantly affecting their quality of life. Research focused on a younger population than traditional OSCC can provide greater understanding of the underlying biology of cancer in young patients and thus help identify new therapies and therapeutic targets. Current information regarding the genetic and epigenetic alterations of OSCC in young patients remains fragmented. Finally, genetic counseling referral for every patient diagnosed with OSCC under 45 years old must be mandatory; family management and risk stratification support prevention and personalize precision treatments impacting in overall survival and life-quality.
